# The Rapidly Changing Patterns in Bacterial Co-Infections Reveal Peaks in Limited Gram Negatives during COVID-19 and Their Sharp Drop Post-Vaccination, Implying Potential Evolution of Co-Protection during Vaccine–Virus–Bacterial Interplay

**DOI:** 10.3390/v16020227

**Published:** 2024-01-31

**Authors:** Kamaleldin B. Said, Ahmed Alsolami, Khalid F. Alshammari, Safia Moussa, Fawaz Alshammeri, Mohammed H. Alghozwi, Sulaiman F. Alshammari, Nawaf F. Alharbi, Amany M. Khalifa, Madiha R. Mahmoud, Kawthar Alshammari, Mohamed E. Ghoniem

**Affiliations:** 1 Department of Pathology and Microbiology, College of Medicine, University of Ha’il, Ha’il 55476, Saudi Arabia; 2 Genomics, Bioinformatics and Systems Biology, Carleton University, 1125 Colonel-By Drive, Ottawa, ON K1S 5B6, Canada; 3 Department of Internal Medicine, College of Medicine, University of Ha’il, Ha’il 55476, Saudi Arabia; 4 Department of Microbiology, King Salman Specialist Hospital, Ha’il 55476, Saudi Arabiadr.kawtharmh@gmail.com (K.A.); 5 Department of Dermatology, College of Medicine, University of Ha’il, Ha’il 55476, Saudi Arabia; 6 Department of Pharmacology, College of Medicine, University of Ha’il, Ha’il 55476, Saudi Arabia; 7 Department of Internal Medicine, Faculty of Medicine, Zagazig University, Zagazig 44519, Egypt

**Keywords:** co-infections, COVID-19 fatality, molecular mimicry, pathogens

## Abstract

SARS-CoV-2 has caused the most devastating pandemic of all time in recent human history. However, there is a serious paucity of high-quality data on aggravating factors and mechanisms of co-infection. This study aimed to identify the trending patterns of bacterial co-infections and types and associated outcomes in three phases of the pandemic. Using quality hospital data, we have investigated the SARS-CoV-2 fatality rates, profiles, and types of bacterial co-infections before, during, and after COVID-19 vaccination. Out of 389 isolates used in different aspects, 298 were examined before and during the pandemic (*n* = 149 before, *n* = 149 during). In this group, death rates were 32% during compared to only 7.4% before the pandemic with significant association (*p*-value = 0.000000075). However, the death rate was 34% in co-infected (*n* = 170) compared to non-co-infected patients (*n* = 128), indicating a highly significant value (*p*-value = 0.00000000000088). However, analysis of patients without other serious respiratory problems (*n* = 28) indicated that among the remaining 270 patients, death occurred in 30% of co-infected patients (*n* = 150) and only 0.8% of non-co-infected (*n* = 120) with a high significant *p*-value = 0.00000000076. The trending patterns of co-infections before, during, and after vaccination showed a significant decline in *Staphylococcus aureus* with concomitant peaks in Gram negatives *n* = 149 before/*n* = 149 during, including *Klebsiella pneumonian* = 11/49 before/during, *E. coli n* = 10/24, *A. baumannii n* = 8/25, *Ps. aeruginosa n* = 5/16, and *S. aureus* 13/1. Nevertheless, in the post-vaccination phase (*n* = 91), gender-specific co-infections were examined for potential differences in susceptibility. Methicillin-resistant *S. aureus* dominated both genders followed by *E. coli* in males and females, with the latter gender showing higher rates of isolations in both species. *Klebsiella pneumoniae* declined to third place in male patients. The drastic decline in *K. pneumoniae* and Gram negatives post-vaccination strongly implied a potential co-protection in vaccines. Future analysis would gain more insights into molecular mimicry.

## 1. Introduction

The recent devasting emergence and re-emergence of infections have reached the highest magnitudes of all time, stimulating an immediate global response [1]. More importantly, the mechanisms of co-infections were not understood. In particular, the types of bacterial pathogens involved and their patterns of infection before, during, and after vaccination were not clear. To understand these mechanisms and potential co-protection by molecular mimicry, we determine the frequencies and most common types of co-infections associated with SARS-CoV-2 before, during, and after mass vaccinations.

The World Health Organization (WHO), the European Union, and the U.S. Centers for Disease Control and Prevention (USA) have prioritized the issue as a threat to human health [2,3,4,5,6]. At present, the annual death estimate is ~3 million humans [7,8]. However, the global cost is expected to be USD 3 trillion by 2050, and 10 million additional people could die each year, costing a cumulative USD 100 trillion [9]. A staggering 8.9 million infections, 33,000 deaths, and an annual healthcare cost of EUR 1 billion in the USA and Europe have been reported [4,5,10]. In the USA alone, another estimate for antimicrobial resistance reported 2,868,700 infections and 35,900 deaths annually [6]. However, the total European cost due to community-acquired infections reached 16.8 billion, with mostly seniors making up 50% of inpatients [11]. This was a significant rise from 2011 in the annual total cost spending in Europe (10.1 billion GBP), including inpatients, outpatient care, and treatment [12]. European countries estimated about 2,609,911 cases and 426,277 claims related to resistant infections alone [13]. The WHO reported a total of 40,000 deaths annually due to nosocomial infections, indicating a rise of 25% in developing countries and 5–10% in developed countries [14,15]. Unfortunately, surveillance is scarce in Middle Eastern countries. Limited estimates were revealed in the following countries: Egypt, followed by Lebanon, Syria, Jordan, Iraq, and the Palestinian territories [16]. Internal instability affected countries, such as Lebanon, where *Acinetobacter baumannii* was the most common pathogen causing a mortality rate exceeding 50% [17]. The Saudi Ministry of Health (MOH) has launched an advanced health cluster system across the country to empower beneficiaries and monitor communicable and noncommunicable diseases [18]. As a result, stricter guidelines and effective control measures are in place [16]. A recent 10-year surveillance in the Arabian Peninsula [19] indicated the emergence of unique infections associated with mortality. Another 5 years of monitoring resulted in increased susceptibility at a tertiary care hospital in Saudi Arabia [20]. However, to the best of our knowledge, there is a serious paucity of high-quality data on the pre- and post-COVID-19 co-infection patterns.

Co-infection rates before COVID-19 vaccination were different in different countries. In China, for example, several studies were conducted with different outcomes on co-infections. Guqin and Zhang showed significantly higher rates of bacterial (25.5%) and fungal co-infections (10.9%) [21]. Similarly, in Jiangsu Province of China, among 257 patients who had confirmed cases of COVID-19, 242 (94.2%) were co-infected with one or more pathogens [22]. Furthermore, in a Hospital in Beijing, 13 patients had positive BAL and there were 73 sputum samples for bacterial cultures, where 56 (58.3%) of them were co-infections [23]. European studies showed a lower rate of co-infections than the previous studies. For instance, in Italy, in a non-survivor population of 16,654 patients, 11% had bacterial or fungal co-infections [21]. Furthermore, the Miulli General Hospital, Italy, examined 233 COVID-19 patients aged 18 to 67 years old and reported that 52 (22.3%) were positive for co-infection [24]. A third Italian study investigated the relationship between SARS-CoV-2 and bacterial and fungal co-infections, where 35 (57%) were positive for bacterial or fungal infections [25]. However, much higher co-infections were reported in other countries, including Middle Eastern countries. For instance, a Palestinian hospital study on COVID-19 patients showed 51.1% of bacterial and 48.9% fungal co-infection [26]. In India, the mortality among patients who developed co-infections was 56.7%, whereas the overall mortality in admitted COVID-19 patients was 10.6%. Gram-negative bacterial isolates were 78%. Another study in India examined 632 patients, and 65 of them (10.3%) had a systemic culture-positive bacterial or fungal co-infection [27,28]. In a Russian hospital, an increase in co-infection by bacterial agents was reported among 433 COVID-19 patients (35.96%) [29]. Similarly, a study on 212 patients revealed 50% mortality in those with fungal- and/or bacterial-positive cultures (*n* = 89; 41.8%) [30]. In a report on 210 patients admitted to an ICU with COVID-19, 55 patients (26%) had positive sterile body fluid cultures for bacteria and fungi [31].

Knowledge of the frequencies and profiles of co-infection after COVID-19 vaccination is crucial in the evaluation of protection and/or co-infections. It has been well established that co-microbial infections aggravate COVID-19, making early detection imperative. High levels of procalcitonin on admission may predict non-survival in critically ill cases in whom bacterial or fungal co-infection is likely [32]. Unfortunately, there are significant variations in the rates of co-infections in different geographic regions globally. A study on 1091 hospitalized COVID-19 patients in Saudi Arabia between March 2020 and December 2020 indicated 70 fatalities overall (6.4%). However, of 182 COVID-19 patients admitted to critical care, 114 patients (62.6%) survived, in-hospital mortality was 13.4%, and the co-infection rate was 67/68 (98.5%), mostly with Gram-negative pathogens [32]. Similarly, a study comprising 76,176 COVID-19 patients estimated that the prevalence of bacterial co-infection was 5.62% [33,34]. Furthermore, a UK study on 6965 COVID-19 patients reported that 8.4% of them had viral co-infections, which is comparatively lower than bacteria [33,34]. However, 55 severe cases and 166 non-severe COVID-19-positive cases concluded that 221 patients had fungal co-infection [35]. An increased rate of mixed microbial co-infections with SARS-CoV-2 was found in 703 COVID-19 patients 75 (10.7%) including 31.5%(17/54) in critical care patients [36]. An intensive care unit study in Iran recorded that 15 out of 73 SARS-COV-2 cases were co-infected by other respiratory pathogens, especially *Candida albicans* and *Klebsiella pneumonia* [37], while a recent study identified 46% (89/191) of patients with co-infection [38]. In Spain, out of 712 COVID-19 patients, 113 (16%) presented bacterial/fungal co-infections or superinfections, and their median age was 73 years [39]. In England, 1% of persons with COVID-19 (2279/223413) had a co-infection/secondary infection, of which >65% were in the bloodstream. Co-infection/secondary bacterial/fungal infections were rare in non-hospitalized and hospitalized persons with COVID-19 and were associated with higher mortality with the most common causative organisms, *Escherichia coli* [40]. The WHO currently recommends against prescribing antimicrobials in mild-to-moderate COVID-19 cases without a clear indication of bacterial infection [41]. A total of 92 out of 1055 (8.7%) patients were positive for respiratory tract infections; however, this type of infection was detected as monomicrobial in 44 patients and as polymicrobial in 17 patients among 61 different patients, 59 (64.1%) male patients, and 33 (35.9%) female patients. Notably, the most resistant bacteria classified as extensively resistant was *A. baumannii*, which was resistant to all antibiotics other than colistin in most reports [42]. Multivariate different independent risk factors for co-infection were evaluated based on their specific treatment strategy [43]. Thus, there is no specific trend in the rates of co-infections after the COVID-19 vaccination campaign in specific countries.

The molecular mimicry between SARS-CoV-2 and other pathogens is a key factor in understating potential mechanisms of co-infections after vaccination. This is true mostly for respiratory pathogens provoking cytokine storms resembling COVID-19 scenarios, such as *S. aureus* [44] and *K. pneumoniae* [45], which react with SARS-CoV-2 spike protein through lipopolysaccharides and induce storm of proinflammatory activity [46,47]. Similarly, it has been shown that several other co-infecting pathogens, including *E. coli* and *A. baumannii*, caused pulmonary injury directly associated with cytokine levels in their infection pattern, which, in turn, were associated with the proliferation of SARS-CoV-2 [48]. It is known that poliovirus, measles virus, dengue virus, and SARS-CoV-2 have high molecular mimicry at the heptapeptide level with the human proteome [22]. Similarly, the proteomes of *BCG*, *Bordetella pertussis*, *Corynebacterium diphtheriae*, *Clostridium tetani*, *Hemophilus influenzae*, *Neisseria meningitidis*, and *Streptococcus pneumoniae* contain numerous potentially cross-reactive epitopes with SARS-CoV-2 [49]. A recent study also reported that the incidence of hepatitis B virus infection among patients with COVID-19 seems to be lower than the incidence of HBV infection in the overall Chinese population. A hypothesis was proposed recently for this phenomenon, arguing that the exhaustion of T lymphocytes may affect HBV-infected patients’ ability to respond to other viruses and then reduce the degree of “cytokine storm”, thus culminating in a less severe disease of COVID-19 [50]. SARS-CoV-2 is associated with *Helicobacter pylori* in the high burden of intestinal metaplasia. This is particularly relevant in *H. pylori*-infected patients because of the increased expression of SARS-CoV-2 entry receptors ACE2 and TMPRSS2 in the affected gastric mucosa, mainly due to the migration of intestine-specific cell types, including enterocytes, within the gastric lining [51]. A viral infection has the ability to dysregulate the immune system, which results in autoimmune diseases such as multiple sclerosis (MS), systemic lupus erythematosus (SLE), and autoimmune hepatitis reported in association with COVID-19 [52,53]. Thus, despite enormous efforts, the patterns, types, frequency, and mechanisms, as well as case fatality rates (CFRs), of co-infections before, during, and after vaccination have not been clear. Thus, the aim of this study was to understand the trending patterns of bacterial co-infections and the frequent types and associated CFRs of each of the three phases of the pandemic. This approach has become imperative as a baseline to understand the mechanisms of microbial co-infections in the COVID-19 background and the potential for molecular mimicry in vaccines.

## 2. Materials and Methods

### 2.1. Microbiological Analysis and Patients’ Demographics

For bacterial co-infection data and microbiological analysis, positive specimens for non-duplicate isolates obtained from clinical infections recovered from hospitals in Ha’il in the periods before, during, and after vaccination were collected. A gap period was considered from the time of vaccine administration (17 December 2020) until the expected significant antibody titer was obtained (April to June 2021), after which all isolates were considered post-vaccination. All isolates before that date were considered before vaccination. Since child vaccination was approved only later during the pandemic, all COVID-19 patients were adults or young adults 18 years and over who were otherwise healthy. For routine microbiology and standard molecular diagnostic methods, specimens were cultured to confirm primary identifications, preparations of inoculums for storage, and automated testing. Automated testing and ID and susceptibility assays were performed on standard diagnostics, such as the BD Phoenix system (BD Biosciences, Franklin Lakes, NJ, USA) and MicroScan plus (Beckman Coulter, Brea, CA, USA). Laboratory records, hospital medical records, and various sources within hospitals were used for data collection on patients’ demographics. This included COVID-19 zones of isolations, patient outcome records in clinical departments, and the results of regional laboratory for COVID-19 diagnosis.

### 2.2. Direct Multi-Gene Molecular Detection of S. aureus Lineages by the GeneXpert System

GeneXpert diagnostics and characterizations were performed by the Cepheid GeneXpert^®^ Dx system using the SA Complete and MRSA assay kits using the manufacturer’s recommendations, names, and codes included in each kit. This system is equipped with multi-gene molecular primers and reagent kits for robust automated direct detection, characterization, and differentiation of different isolates. This test utilizes automated real-time polymerase chain reaction (PCR). Confirmatory susceptibility assays were carried out by culturing. GeneXpert Dx is an all-in-one system that integrates sample purification, nucleic acid amplification, and detection of the target sequence in simple or complex samples using real-time PCR. It consists of an instrument, a personal computer, and preloaded software for running tests and viewing the results. A single-use disposable self-contained cartridge with PCR reagents is inserted and inoculated directly with swabs/samples. In addition to avoiding environmental cues that alter the genome, cross-contamination between samples or during specimen collection or processing, as well as cross-sequence contaminations in molecular tests, are all remote since the cartridge is a disposable, closed, and self-contained kit. A sample processing control (SPC) and a Probe Check Control (PCC) are also included. The SPC is present to control for adequate processing of the target bacteria and to monitor the presence of inhibitor(s) in the PCR reaction. The PCC verifies reagent rehydration, PCR tube filling in the cartridge, probe integrity, and dye stability.

### 2.3. Statistical Analysis

Collected data were analyzed using Statistical Package for Social Sciences software (IBM SPSS; Version 24 SPSS version 23.0 for Windows (SPSS, Inc., Chicago, IL, USA). Descriptive and stratified analyses were conducted; we present absolute numbers, proportions, and graphical distributions. We conducted exact statistical tests for proportions and showed *p*-values where appropriate (a *p*-value < 0.05 was considered statistically significant).

## 3. Results

**Figure 1:**
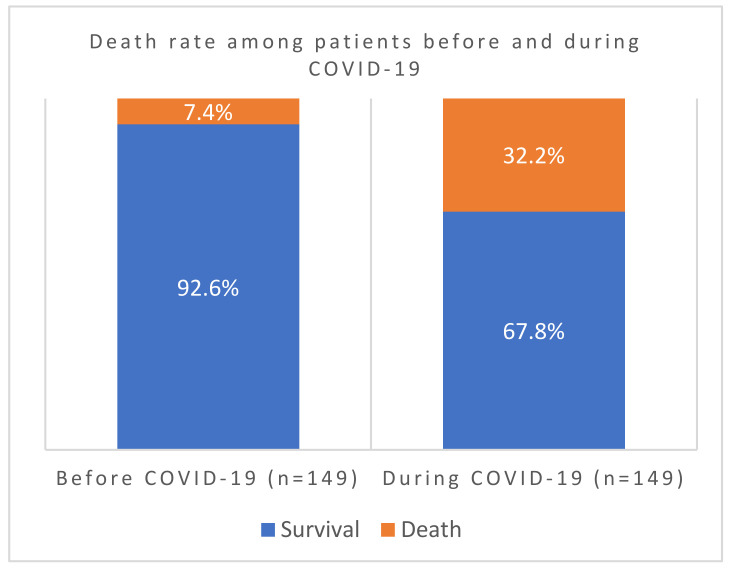
Death rates before and during COVID-19 in Ha’il hospitals, Saudi Arabia.

In this comprehensive study, we have investigated 389 cases for clinical profiles, case fatality rates, and patterns of bacterial co-infections before, during, and after COVID-19 vaccination. We tried to understand factors that aggravate the disease and the potential mechanisms during host–bacteria–viral interplay. We have screened out all confounding factors that may have an influence, including other existing respiratory syndromes, age, and gender-specific factors of patients admitted before and during the pandemic. As indicated in Figure 1, out of the 298 patients screened, the COVID-19 case fatality rate during the pandemic was 32.2% compared to only 7% before. The association of case fatality to the pandemic was significantly higher during than before COVID-19 (*p*-value = 0.000000075).

**Figure 2:**
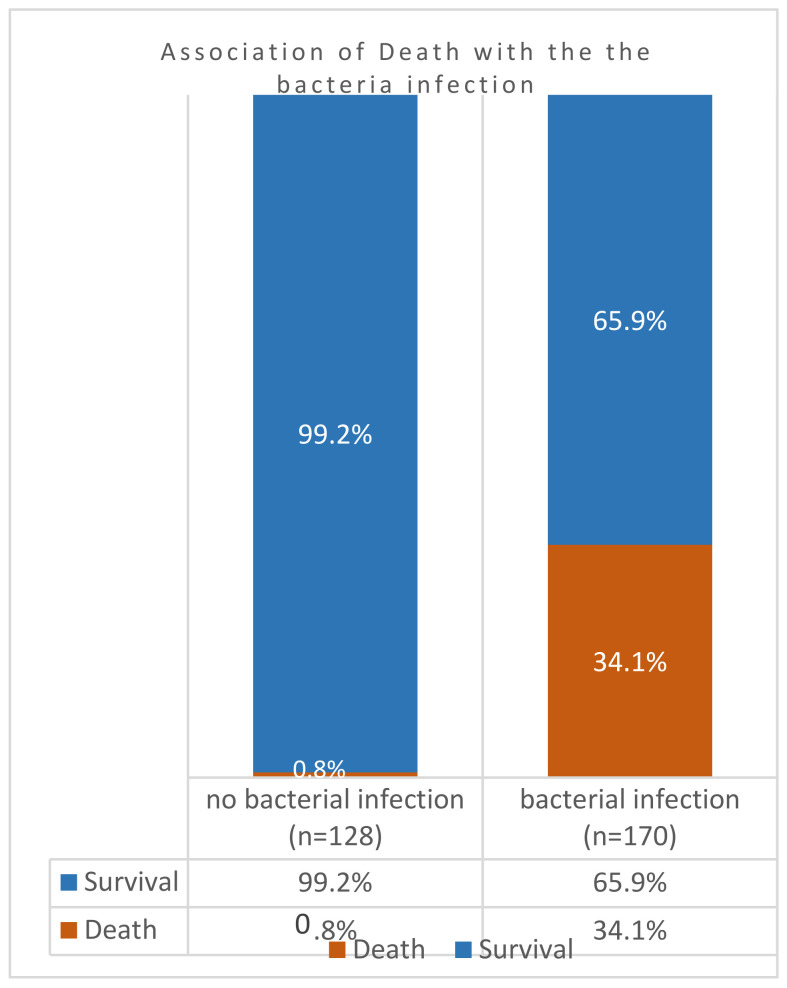
COVID-19 case fatality rates among co-infected and non-co-infected patients in Ha’il Hospitals, Saudi Arabia.

**Figure 3:**
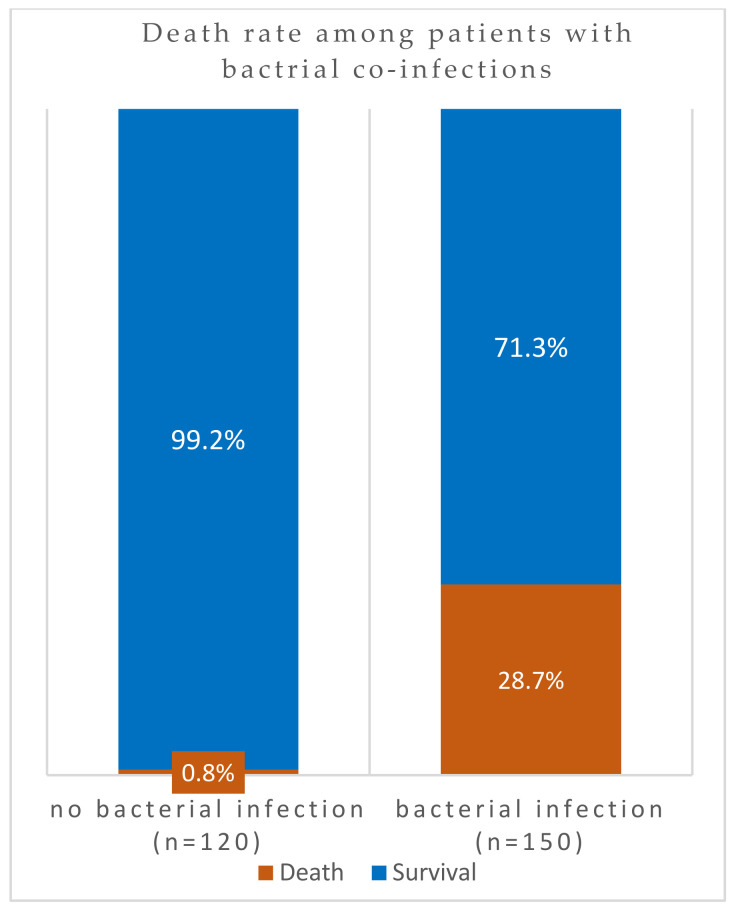
Death rates among co-infected COVID-19 patients without underlying respiratory syndrome in Ha’il, Saudi Arabia.

However, in 298 patients, a comparison of case fatality rates among co-infected COVID-19 patients (*n* = 170) against those without co-infection (*n* = 128) indicated that the death rates were significantly higher (34%) in the former group (Figure 2). The association of mortality and case aggravation to co-infection was significantly higher, as indicated by the *p*-value = 0.00000000000088. In other words, almost 100% (99%) of patients without SARS-CoV-2 superinfection survived the pandemic. However, the exclusion of all patients with Severe Respiratory Distress Syndromes in patients with bacterial co-infections also resulted in higher levels of mortalities (Figure 3). Among these patients without underlying respiratory syndrome (*n* = 270), bacterial infection was associated with a higher death rate, as shown by the highly significant value (*p*-value = 0.00000000076).

**Figure 4:**
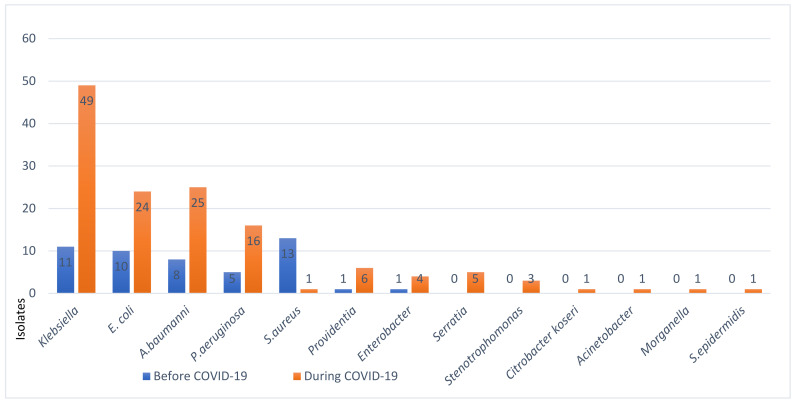
Bacterial isolates and co-infections before and during COVID-19 in Ha’il, Saudi Arabia.

**Figure 5:**
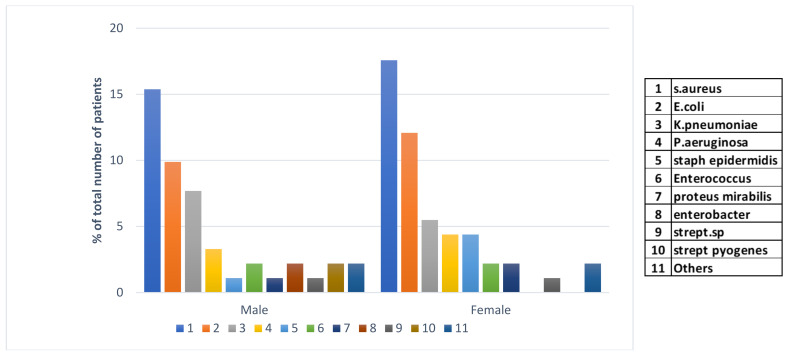
Gender-specific bacterial co-infections after COVID-19 vaccination campaigns in Ha’il, Saudi Arabia.

Pathogenic populations of microbial co-infections with SARS-CoV-2 presented with significant changes in their types and profiles. To understand this important factor in host–viral–bacterial interactions, we have examined the trending patterns of infections across three phases of the pandemic, i.e., before, during (Figure 4), and after vaccination (Figure 5). The following frequency of major co-infections were found (*n* = 149 before/*n* = 149 during): *Klebsiella pneumonia* (*n* = 11/49 before/during; *E. coli n* = 10/24, *A. baumannii n* = 8/25, *Ps. aeruginosa n* = 5/16, and *S. aureus* 13/1. The major findings were a significant decline in the rates of Gram-positive species, mainly *Staphylococcus aureus*, while a steady increase in a few Gram-negative species was observed during the pandemic (Figure 4).

The major finding of this investigation was in the peaks in types of bacterial pathogens co-infecting with the SARS-CoV-2 virus during and after COVID vaccination. In the Ha’il region, a 100% vaccination rate was achieved during the early stage of the vaccine campaigns consisting of Pfizer (New York, NY, USA), Moderna (Cambridge, MA, USA), and the Oxford/AstraZeneca (Cambridge, UK) recombinant vaccine. In 91 bacterial co-infection cases post-vaccination, we have examined gender specificity to account for potential differences in susceptibility, unlike before the pandemic. Methicillin-resistant *S. aureus* (MRSA) was the dominant hospital pathogen isolated from cases of infections in both genders after COVID-19 (Figure 5). This was followed by *E. coli* in males and females, with the latter gender showing higher rates of isolations in both species. *Klebsiella pneumoniae* in third place was isolated from male patients after vaccination. Other Gram-negative and -positive pathogens presented with a lower rate of isolation. A much lower frequency of bacterial isolations was reported after the COVID-19 pandemic compared to before and during the pandemic.

## 4. Discussion

In the current study, we have investigated the factors that exacerbate COVID-19 pandemic fatality rates. By examining patterns of infections across three phases of the COVID-19 pandemic, i.e., before, during, and after vaccination, we have also identified missing gaps of potentially novel mechanisms that influenced the patterns of co-infection during host–bacteria–viral interplay. In agreement with the widely reported finding, the higher case fatality rates significantly associated with COVID-19 (32.2%), (*p*-value = 0.000000075) compared to before the pandemic indicated enhanced virulence and epidemicity of the virus. The *Lancet* Commission on lessons for the future from the COVID-19 pandemic has described the staggering death toll of COVID-19 as both a profound tragedy and a massive global failure at multiple levels [54]. However, the global case fatality rate of COVID-19 has decreased by 96.8% during the last years of the pandemic [55]. Intriguingly, the unprecedented sharp increase followed by the rapid decline of the pandemic after vaccination has never been witnessed in modern human history. As with all pandemics, the aftermath of SARS-CoV-2 has left several novel observations on its epidemicity, virulence, and mechanisms of co-infections. We have found that while *S. aureus* dominated before and after vaccination, Gram-negative pathogens, *K. pneumoniae*, *E. coli*, *A. baumannii*, and *Ps. Aeruginosa*, peaked in the middle phase during but before vaccination. It is plausible that these observations provide proof of concepts about two potential mechanisms during and after the vaccination phase. An important gap exists during but before the vaccination phase; it is not clear whether both species could have used a common mechanism to elicit a cytokine storm or whether the selective and rapid outgrowth of *K. pneumoniae* might have suppressed *S. aureus.* The latter species produces potent exoproteins and excretes several toxins to induce cytokine storms without the need for cell suppression, while Gram negatives use cell-bound LPS, which explains the need for cell concentrations. Thus, future vertical studies would gain more insights into the mechanisms of co-infections with SARS-CoV-2.

The uniquely trending pattern of SARS-CoV-2 co-infection with bacterial pathogens in the three phases of the pandemic, namely before, during, and after vaccination, has left a remarkable phenomenon. Although co-infections are widely reported as major aggravators, the mechanisms of how they occur are poorly understood. The major finding in this study was the sudden drop in the frequency of isolation of *S. aureus* lineages during the pandemic pre-vaccinations, whereas a steady increase in limited Gram-negative pathogens was observed at the same time during this phase. Bacterial co-infectors mostly included *Klebsiella pneumonia*, *E. coli*, *A. baumannii*, and *Ps. Aeruginosa*, in that order. This was followed by another peak of *S. aureus* infections toward the aftermath of the pandemic right after vaccination dominated all Gram-negative pathogens (Figure 5). *Staphylococcus aureus* is a very well-known superbug that elicited a massive cytokine storm, leading to a serous necrotizing pneumonia outbreak reported in the CA-MRSA pandemic a decade ago [56]. In addition, a recent experimental demonstration proved that *S. aureus* provoked a cytokine storm in BALB/c mice [44]. Nevertheless, recent experimental data from BALB/cJ mice indicated that co-infected mice showed a massive immune storm and severe clinical disease, leading to a higher mortality rate within 48 h of *K. pneumoniae* infection. Significantly higher bacterial loads in the lungs were observed, albeit viral loads remained unchanged between co-infected and single-infected mice [45]. It is interesting that these two species provoked cytokine storms during lung necrotizing infections; however, it remains to be seen whether they both use the same mechanism of induction of major histocompatibility complex (MHC) class II on antigen-presenting cells (APCs). A highly significant clue for a common induction is the pattern of co-infection observed in this study. We have observed that all *S. aureus* lineages, including methicillin-sensitive, MRSA, and CA-MRSA, as well as animal lineage rates, drastically reduced during COVID-19 before vaccination and then peaked right after vaccination. If this was a competitive overgrowth by Gram negatives occupying cytokine induction sites on APCs, then it is difficult to explain their sharp decline after vaccination where *S. aureus* peaked. It is plausible that there is an element of potential molecular mimicry with Gram negatives in the vaccines. In support of this, a case of a community-acquired MRSA necrotizing lung infection occurred right after recovery from SARS-CoV-2 infection [57]. In addition, evidence demonstrated that SARS-CoV-2 spike protein serves as a lipopolysaccharide delivery system and binds to bacterial LPS, boosting an overzealous storm of proinflammatory activity [46,47]. Similarly, it has been shown that several other co-infecting pathogens, including *E. coli* and *A. baumannii*, caused pulmonary injury directly associated with cytokine levels in their infection pattern, which, in turn, were associated with the proliferation of SARS-CoV-2 [48]. Although there are several scenarios in the molecular mechanisms of co-infections, this study provides clear observations about the coexistence patterns of different co-infecting pathogens in COVID-19 backgrounds.

## 5. Conclusions

Thus, we have investigated all three phases of SARS-CoV-2 pandemic patterns of infection, i.e., before, during, and after vaccination. In this study, we provide factors that aggravated COVID-19 disease fatality rates, including patient gender and bacterial co-infections. The high significant rates of mortality in co-infected patients during COVID-19 indicated the influence of bacterial pathogens in patients’ worse outcomes. Intrudingly, the positive selection for co-infection by limited Gram negatives during COVID-19 with a concomitant decline in *S. aureus* followed by peaks of the latter and drastic decline of the former species in the post-vaccination phase strongly implied the potential element of molecular mimicry in the vaccine component. Future molecular analysis of host–virus–bacterial interplay for identification and characterization of common gene candidate(s) involved in cytokine storms has become imperative since there is a pandemic outbreak of hypervirulent Gram negatives and CA-MRSA necrotizing pneumonia. The project is limited by the lack of wide regional coverage that could provide larger sample sizes for more insights into the mechanisms of pathogenicity and virulence. The limitation of this study is that the number of post-vaccination samples were less than before because the infection rates sharply declined. In addition, a large cohort study comprising different locations in the region would have gained more insights.

## Data Availability

There is no additional data deposited on any other site other than those in this manuscript.
